# Prevalence of Pneumonia in Sheep and Goats Slaughtered at Elfora Bishoftu Export Abattoir, Ethiopia: A Pathological Investigation

**DOI:** 10.1155/2019/5169040

**Published:** 2019-07-18

**Authors:** Berhanu Mekibib, Tadesse Mikir, Amene Fekadu, Rahmeto Abebe

**Affiliations:** ^1^Faculty of Veterinary Medicine, Hawassa University, P.O. Box 05, Hawassa, Ethiopia; ^2^Field Veterinarian, Ministry of Agriculture and Natural Resources, Amhara Region, Ethiopia

## Abstract

Accurate clinical diagnosis of pneumonia, the leading cause of mortality in small ruminants, is difficult and usually requires postmortem examination of the lungs. An active abattoir survey was conducted between November 2017 and April 2018 to estimate the prevalence and characterize the gross and histopathological lesions of pneumonic lungs in 864 clinically healthy young small ruminants (490 sheep and 374 goats aged 1.5 to 3 years) raised for meat in different parts of the country and slaughtered at Elfora Bishoftu export abattoir, Ethiopia. Out of the total lungs examined grossly, pneumonic lesions were found in 158 (18.29%) lungs. On histopathological examination of the lungs with gross pneumonic lesion, however, typical pneumonic lesions were diagnosed in 148 (17.13%) lungs only. No significant (p>0.05) difference was noted in the prevalence of pneumonia between sheep (17.14%) and goats (17.11%) in histopathological examination. Based on the predominant histopathological findings, the pneumonic lesions were characterized as interstitial pneumonia (41.9%), acute suppurative bronchopneumonia (25.7%), acute fibrinous bronchopneumonia (24.3%), chronic bronchopneumonia (6.1%), aspiration pneumonia (4.7%), bronchointerstitial pneumonia (3.4%), and ovine pulmonary adenomatosis (3.4%). The study further showed the spread of ovine pulmonary adenomatosis and ovine progressive pneumonia (Maedi) from the central highlands to areas that were previously free from these diseases. Due to its better diagnostic capacity, histopathology should be employed routinely as an ancillary test in the major abattoirs and regional veterinary laboratories to generate additional epidemiological data for a better disease control and prevention measures. Further studies are also recommended to identify the etiological agents of pneumonia in sheep and goats and thereby to formulate feasible and cost-effective interventions.

## 1. Introduction

Ethiopia has 30.7 million sheep and 30.2 million goats [1]. Despite this huge population, the country is still unable to meet the growing domestic and export need for mutton and chevon because of several reasons. Poor management and husbandry practices and diseases of varied etiologies are among the leading bottlenecks of sheep and goat production [2]. Among the wide range of diseases that affect sheep and goats, respiratory diseases are most frequent as air and blood are their main routes of transmission [3]. Overcrowding coupled with the involuntary inhalation of air polluted with a variety of potentially injurious materials is the most important risk factor for transmission [4].

Lungs are the most exposed organs to different aggressions because of their anatomical and histological particularities [4–6]. The extensive surface area and the delicate vascular bed in the lungs are known to expose the lungs for infection with several pathogens [4]. Pneumonia, a respiratory disease arising from an inflammatory response of the lung parenchyma, is still the major disease limiting the development of animal production in the tropics [7]. In Ethiopia, it is the leading health problem of small ruminants and causes huge financial losses through morbidity and mortality [2, 8, 9].

Pneumonia is regarded as a disease complex, involving interactions between the host (immunological and physiological), multiple etiological agents (viral, bacterial, mycoplasmal, parasitic, etc.), and environmental factors (temperature, humidity, dust levels, etc.) [10, 11]. Irrespective of the cause, respiratory diseases significantly impact upon the profitability of farms, both directly and indirectly [12] and compromise the welfare of the animals [13]. In general, respiratory ailments are the major cause of death in lambs and decreased productivity in older animals in most developing countries [6].

Clinical diagnosis of pneumonia is difficult and usually involves physical examination, imaging, serology and identification of the aetiological agent from nasal swabs, bronchial lavages, and even feces (for verminous pneumonia) [4, 13, 14]. However, these attempts should be complemented with postmortem examination of the lungs either at necropsy or slaughter for accurate diagnosis [6, 12, 14]. Moreover, because of the poor veterinary services in most parts of Ethiopia, majority of the sheep and goats brought to the abattoir for slaughter may harbor chronic or subclinical infections which are rarely detected during antemortem examination [15]. Therefore, apart from its primary role, an abattoir can also be utilized as an easy and cheap source of data for evaluation of the epidemiological aspects of animal diseases including pneumonia [16, 17].

In Ethiopia, some studies have reported that pneumonia is among the major causes of lung condemnation in sheep and goats slaughtered at different abattoirs in the country. According to most recent studies pneumonia was responsible for condemnation of 32.2% and 60% of lungs examined at Addis Ababa [18] and Elfora Bishoftu export abattoir [19], respectively. However, to the authors' knowledge, very little work has been done to date to characterize the different forms of pneumonia histopathologically. Thus, the present study was conducted with the objective of estimating prevalence and characterizing the gross and histopathological lesions of pneumonic lungs in sheep and goats slaughtered at Elfora Bishoftu export abattoir.

## 2. Materials and Methods

### 2.1. Study Area

**Figure 1:**
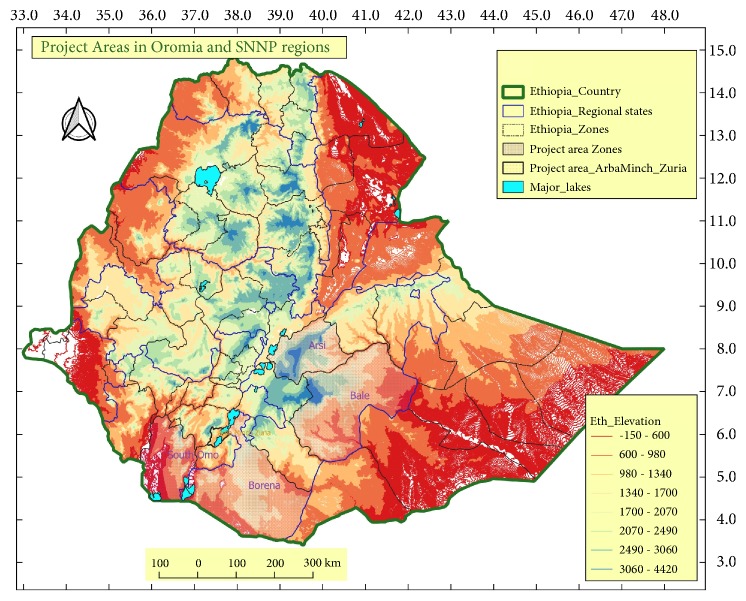
Map of the study area showing the elevation and agroclimatic zones.

The study was conducted between November 2017 and April 2018 in Elfora Bishoftu export abattoir, which is located at about 45 kilometers South East of Addis Ababa, the capital city of Ethiopia. It is one of the abattoirs which export meat to Saudi Arabia, Turkey, Egypt and United Arab Emirates. Most of the sheep and goats slaughtered in the abattoir and included in this study originated predominantly from Arsi - Bale highlands and the lowland areas of the country such as Borana zone, south Omo zone, and Arba Minch zuria district (Figure 1).

Arsi-Bale highlands are found in the Oromiya Regional State southeast of Ethiopia. The area majorly represents the central plateau (2000-2500 meters above sea level) and receives a bimodal rainfall occurring from July to October and April to May. The average annual temperature and rain fall ranges between 12-18°C and 900-1400mm, respectively [20].

Arba Minch zuria district is located in southern Ethiopia at about 500 km from Addis Ababa. The area has an altitude ranging from 1300 to 2600m a.s.l. with mean annual rainfall of 900-1000mm and mean annual temperature of 23°C. Therefore, the area has a subhumid climate with moderately hot temperature. The area is characterized by wooded grasslands and riparian vegetation [21].

South Omo zone is located in Southern Nations, Nationalities and People's Regional state (SNNP). The temperature of the area falls between 15.7°C and 38°C. The zone is located in 4.43^0^ – 6.46^0^ North latitude and 35.79^0^-36.06^0^ East longitude. The climatic condition ranges from Dega (humid) to Kola (semiarid) which constituted 34.4% of the zonal climatic condition [22].

Borana zone is situated at about 600 kms South of Addis Ababa. The area is bordered by Kenya from south, Somali regional state from east, highlands of Guji from the north and Southern Nation Nationalities, and People Regional State from the west [23]. The climate is generally semiarid with annual average rainfall ranging from 300mm in the south to over 700mm in the north. Annual mean daily temperature varies from 19°C to 24°C with moderate seasonal variation. Season affects herding patterns due to its effect on forage and water resources availability [23].

### 2.2. Study Animals

All the sheep and goats brought to the abattoir were young indigenous breeds raised for meat and milk (goats) production and managed under extensive production system either as part of a mixed crop–livestock production system (in highland areas) or a pastoral system of production (in lowland areas). Sheep and goats are usually kept mixed with other livestock species (cattle, camel, and donkeys) in communal grazing and watering areas. For the purpose of export, they were purchased from different local markets and then transported to the abattoir by lorries and on foot. In the abattoir they were fed, watered, and rested for about 5 to 7 days before slaughter.

### 2.3. Sample Size and Sampling Procedure

The sample size required for this cross-sectional study was calculated for each species using the formula given by Thrusfield [24]. Using 50% expected prevalence, 95% confidence level, and 5% desired precision, a total of 864 small ruminants (490 sheep and 374 goats) were included in the study. The samplings were done three days per week and on average 30 small ruminants (out of 290 to 320 slaughtered) were sampled and examined each day. These animals were selected by a systematic random sampling whereby every 10th animal walking into the lairage was selected and marked. All study animals were male, local breeds and had medium body conditions. The study animals were aged 1.5 to 3 years as estimated by means of dentition as described for African indigenous goats [25]. For detail histopathological examination, lungs with pneumonic lesions were selected purposively.

### 2.4. Methodology

#### 2.4.1. Gross Examination and Sample Collection

At the abattoir, routine meat inspection was conducted by the local meat inspectors (led by a veterinarian) according to procedures recommended by Gracey et al. [26]. Lungs of the selected study animals suspected of pneumonia were collected and thoroughly inspected by the first and third authors for the presence of different lesions using visualization, palpation, and multiple systemic incisions when and wherever required. The texture, consistency, color, adhesion, pattern, distribution and number of the lesion(s), the nature of bronchial exudates if any, and the lobe(s) involved were recorded on a format prepared for this purpose. Representative pieces of tissues (4-5 mm in thickness) were then taken from the pneumonic lungs, fixed with 10% neutral buffered formalin, and transported to Hawassa University, Veterinary Pathology and Parasitology Laboratory.

#### 2.4.2. Histopathological Examination

Following at least 48 hrs of fixation, representative tissue samples were properly trimmed, labeled, and processed by a routine paraffin-embedded technique. Briefly, the fixed tissue samples were cut into pieces of 2–3 mm thickness and washed thoroughly with water for several hours before putting in ascending grades of alcohol for dehydration, followed by clearance in xylene and embedded in paraffin. Sections of 4–5 micron thickness were cut and stained by Harri's hematoxylin and Eosin method [27]. Finally the stained slides were examined systematically at 10X and 100X magnifications for the presence of characteristic and/or suggestive lesions using ordinary light microscope. The different forms of pneumonia were then classified according to the involvement of pulmonary regions and anatomical sites and nature of the inflammatory exudate and reaction present [28, 29].

### 2.5. Data Management and Analysis

Data obtained during antemortem, gross, and histopathological examinations were recorded on Microsoft excel spread sheet. The difference in the prevalence of pneumonia between sheep and goats was analyzed using chi-square independent test. A p value < 0.05 was considered statistically significant.

## 3. Results

Out of the total of 864 small ruminants' lungs subjected to gross examination, pneumonic lesions were detected in the lungs of 158 (18.29%) animals. However, on histopathological examination of the suspected lesions, pneumonia was diagnosed in 148 (17.13%) animals only. The pneumonia suspected in the lungs of the other 10 animals during gross examination was histopathologically confirmed to be nonpneumonic pneumopathies, namely, hemorrhage (n=8) and atelectasis (n=2). The prevalence of pneumonia was quite similar in both sheep and goats (p>0.05) (Table 1).

On the basis of predominant histopathological findings, the pneumonic lesions were further classified into interstitial pneumonia, acute suppurative bronchopneumonia, acute fibrinous bronchopneumonia, chronic bronchopneumonia, aspiration pneumonia, bronchointerstitial pneumonia, and ovine pulmonary adenomatosis. The three forms of bronchopneumonia altogether accounted for 56.1% (n = 83) of the pneumonic lungs observed. Of the total pneumonic animals, six animals were affected with two types of pneumonia while four animals were with three forms (Table 2).

### 3.1. Interstitial Pneumonia

**Figure 2:**
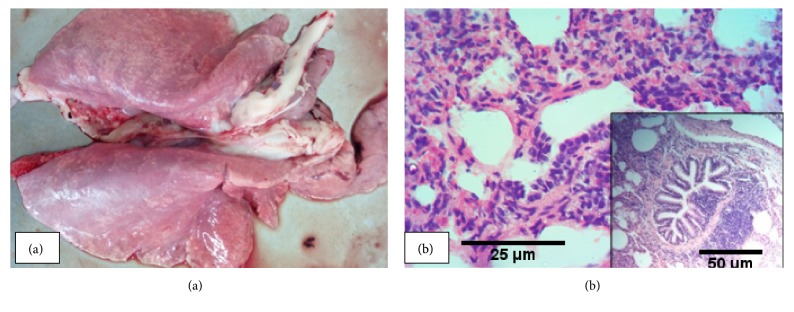
*Interstitial pneumonia*: enlarged lungs with rib imprint, rubbery in consistency, and meaty in appearance (a) and thickening of the interalveolar septa because of proliferation of smooth muscle and fibroblasts along with inflammatory cells infiltration (b).

Interstitial pneumonia was encountered in 41.9% of the animals with gross pneumonic lesions. Grossly, most parts of the lungs were enlarged, rubbery in consistency and meaty in appearance (Figure 2(a)). The lungs had rib imprints and the lesions were generalized but predominantly dispersed on the caudal aspect of the lungs. Microscopically, the lesions were characterized by thickening of the interalveolar septa because of proliferation of smooth muscle and fibroblasts along with infiltration with lymphocytes, neutrophils, and macrophages (Figure 2(b)). Moreover, lymphofollicular proliferation with follicle-like aggregations, peribronchial and perivascular cuffing (Figure 2(b) Inset) and thrombus were noted in 45% of the cases with interstitial pneumonia. Unlike the bronchopneumonia cases, there was no obvious exudate in the alveolar spaces and airways. This form of pneumonia was also seen concurrently with acute fibrinous bronchopneumonia (n=5) and acute suppurative bronchopneumonia (n=1).

### 3.2. Acute Suppurative Bronchopneumonia

**Figure 3:**
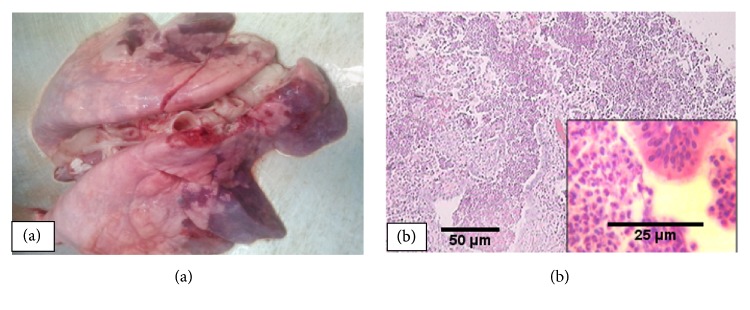
*Acute suppurative bronchopneumonia: *red to gray cranioventral lobes (a) with characteristic polymorphonuclear cells and few mononuclear cells infiltration in the alveoli, bronchi, and bronchioles (b).

Acute suppurative bronchopneumonia (ASBp) was detected in 25.7% of the pneumonic animals. Grossly the lungs were red to gray in color, consolidated in consistency, and sometimes caeseous upon incision. The lesions were mainly distributed to the cranioventral aspects of the lungs (Figure 3(a)). Histopathologically, the suppurative areas were characterized by infiltration of polymorphonuclear cells (mainly neutrophils) and few mononuclear cells in the alveoli, bronchi, and bronchioles (Figure 3(b)). Sloughed epithelial cells and necrotic debris were also noted in the bronchi and bronchioles. Moreover, bacterial colonies were noticed in the alveoli of some cases.

### 3.3. Acute Fibrinous Bronchopneumonia

**Figure 4:**
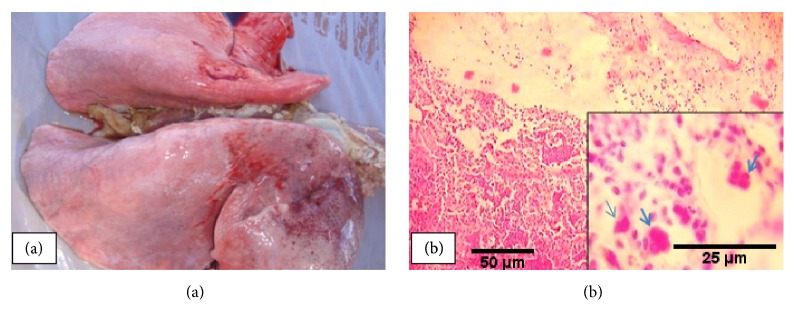
*Acute fibrinous bronchopneumonia*: consolidation and mottling on cranial lobes (a) with marked fibrinopurulent exudates and syncytial cells (arrow) in the alveoli, bronchi, and bronchioles (b).

Acute fibrinous bronchopneumonia (AFBp) was observed in 24.3% of the affected animals. The lesions were grossly characterized by areas of consolidation mostly in the cranial, cardiac, and accessory lobes (Figure 4(a)). The lungs were hard to cut and some cases showed marbling with thickening of interlobular septa and pleura. Histopathologically, the lesions were characterized by exudation in the bronchi, bronchioles, and alveoli majorly composed of fibrin and neutrophils, denuded epithelial cells, and necrotic debris. Interlobular septa were thickened with infiltration of fibrinocellular exudates comprising predominantly of neutrophils with occasional mononuclear cells (Figure 4(b)). In some (n=11) cases, desquamation of epithelial cells, spindle-shaped cells, oat streaming cells, and syncytial cells were also noted.

### 3.4. Chronic Bronchopneumonia

**Figure 5:**
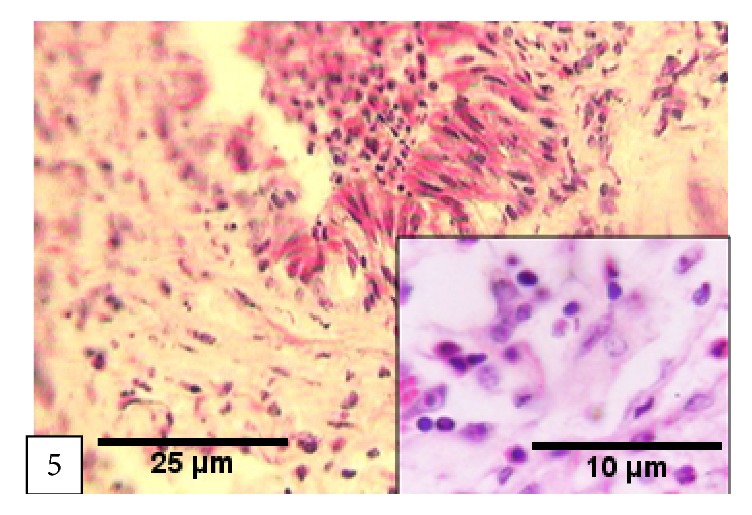
*Chronic bronchopneumonia*: extensive fibrinous thickening of the pleura and varying degree of mononuclear cells infiltration in the alveoli.

Chronic bronchopneumonia (CBp) was seen in 6.1% of the affected animals. It mainly involved the cranial lobes, and the lesions in the affected parts were firm to hard in consistency and pale to gray in color. The pleura were shriveled and adhered to the chest wall in three cases. Microscopically, extensive fibrinous thickening of the pleura and varying degree of mononuclear cells infiltration in the alveoli were observed (Figure 5).

### 3.5. Bronchointerstitial Pneumonia

**Figure 6:**
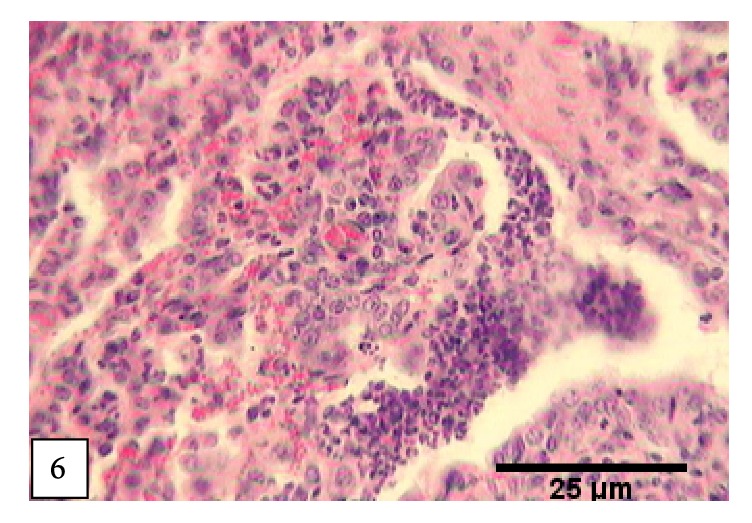
*Bronchointerstitial pneumonia: *thickening of intra-alveolar septa with prominent exudate in the alveoli and respiratory tree.

The lesions suggestive of bronchointerstitial pneumonia were observed in 3.4% of the affected animals (2 sheep and 3 goats). Grossly, the lesions were not remarkable but the affected parts were meaty in appearance, uncollapsed, and distributed on both the anterior and caudal lobes. Microscopically, the characteristic features of both suppurative bronchopneumonia and interstitial pneumonia were found admixed (Figure 6).

### 3.6. Aspiration Pneumonia

**Figure 7:**
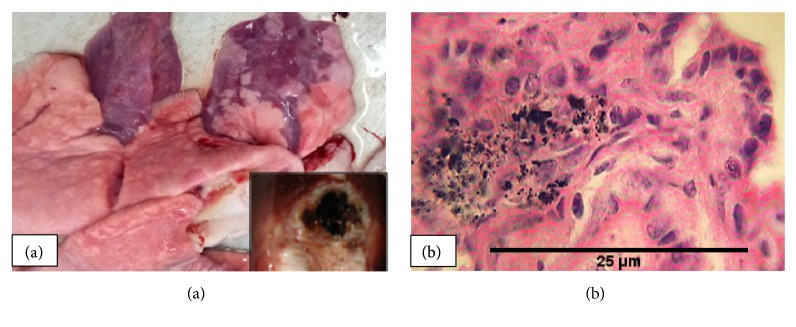
*Aspiration pneumonia*: atelectasis on the cranioventral lobes with green-brown contents in the air ways (a) suggesting foreign body granuloma (b).

Microscopic lesions typical of aspiration pneumonia were detected in 4.7% of the affected animals. Of these animals, four had prominent atelectasis mainly on the cranioventral aspect of their lungs. Grossly, the affected portions of the lungs were congested and somewhat meaty in consistency. Moreover, gray to green-brown foul-smelling exudates were expressed from small airways upon compression in three lungs (Figure 7(a)). Microscopically, the lesions were ranging from bronchopneumonia to granulomatous in type but all were characterized by the presence of aspirated/foreign material in the bronchi and bronchioles (Figure 7(b)).

### 3.7. Ovine Pulmonary Adenomatosis (OPA)

**Figure 8:**
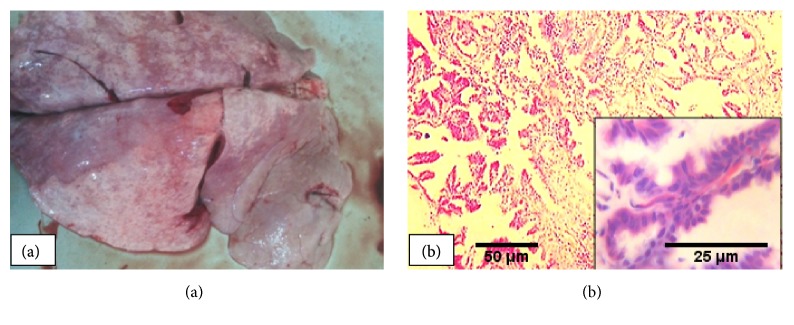
*Ovine Pulmonary adenomatosis*: enlarged, heavier, grayish to white granular masses with meaty to firm in consistency (a) with characteristic papillomatous proliferation of type II pneumocytes that project into the alveoli (b).

Ovine pulmonary adenomatosis (OPA) was found in 3.4% of pneumonic animals, all of which were sheep. It was found as concurrent infection with fibrinous bronchopneumonia and ovine progressive pneumonia, one case each. Grossly, the lungs were enlarged, heavier, grayish to white granular masses with meaty to firm in consistency (Figure 8(a)). Moreover, grayish exudates were appreciated from cut surfaces of variably sized elevated nodules. Microscopically, the lesions were characterized by papillomatous proliferation of type II pneumocytes that project into the alveoli (Figure 8(b)). Apart from the adenomatous appearance, neutrophilic aggregates were also observed in the lumen of alveoli, bronchioles, and bronchi of 1 lung and characteristic lesion in others.

## 4. Discussion

Respiratory diseases, particularly pneumonia, constitute significant economic losses after diarrhea in small ruminants [30]. In Ethiopia, respiratory diseases of sheep caused by concurrent infections have been identified as the leading health problem accounting for more than half of the overall mortality of sheep in the central highlands [8]. The current study revealed that 17.14% of sheep and 17.11% of goats examined at an export abattoir had pneumonic lesions showing that the condition is an important cause of organ condemnation and thereby economic losses.

In the present study, bronchopneumonia in the form of acute suppurative, acute fibrinous, and chronic bronchopneumonia accounted for the greatest proportion (56.1%) of pneumonia encountered. In agreement with the present finding, previous investigations in other countries also reported bronchopneumonia as the commonest type of pneumonia in sheep and goats [28, 29, 31, 32]. The gross and histological lesions of the different forms of bronchopneumonia were consistent with the findings of other studies [11, 33–36]. The lesions could be associated with contagious caprine pleuropneumonia (in goats) or pneumonic pasteurellosis as these diseases are characterized by fibrinous and suppurative bronchopneumonia patterns [4, 37, 38]. These specific pneumonic diseases were also reported to be prevalent in some parts of Ethiopia [39–41]. The majority (89.2%) of bronchopneumonia noted in the current study was acute type. This suggests that the disease was most likely attributed to exposure of the animals to various stresses during transportation for slaughter. Stress factors like long transportation under poor condition, overcrowding, sudden climate change at transit, unrest in the lairage, inadequate feed and water provision, and other underling diseases including predisposing viral infection of the respiratory system such as parainfluenza-3, adenoviruses, and respiratory syncytial virus are widely accepted as predisposing factors for bacterial pneumonia [4, 42].

Although bronchopneumonia is multifactorial in origin, it is mainly caused by bacterial pathogens, namely,* Mannheimia haemolytica, Pasteurella multocida, Histophilus somni, *and* Mycoplasma *spp. when the immune system of the animal is compromised by aforementioned stress factors [4, 42]. The above bacteria and other opportunistic bacteria were isolated from the respiratory tract of healthy and pneumonic sheep and goats in different parts of the country [43–48].

Interstitial pneumonia was the second dominant type of pneumonia in this study detected in 41.9% of the pneumonic animals. The gross and histopathological characteristics of the lesions in the affected lungs were similar to earlier reports [11, 49, 50]. It was appreciated in both species; however, the proportion was considerably higher in sheep (51.2 %) than in goats (29.7 %). Although some environmental factors are rarely incriminated, interstitial pneumonia in small ruminants is mainly caused by two related viral pathogens, namely, ovine lentivirus (OvLV) in sheep and Caprine arthritis-encephalitis virus (CAEV) in goats, and usually referred as lentiviral pneumonia [4]. Unless complicated with other pathogens, this form of pneumonia is usually subclinical and only detected during histopathology [51]. Ovine progressive pneumonia (also known as Maedi), a chronic disease of sheep caused by OvLV, has been reported from different parts of the country through serological, histopathological, and clinical studies [44, 52–58]. According to the studies, the seroprevalence of ovine progressive pneumonia in Ethiopia ranges from 5.4 % to 74%. Following its first report in 1991 in Agarfa and Arsi Rural Development Unit (ARDU) exotic sheep breeding farms/centers, there is a growing evidence for the dissemination of the disease to several parts of the country through the distribution of exotic or cross breed rams from the breeding and multiplication center [54]. Although lentiviral pneumonia was not reported previously in goats in the country, the interstitial pneumonia observed in this species might be caused by Caprine arthritis-encephalitis virus (CAEV) and/or Maedi-Visna virus infection. While further confirmatory research is needed, the authors suspect the introduction of the strains into local goats following the attempt made during the past one decade to improve the productivity of the local goat population by importing exotic goat breeds. Moreover, the greater homology of CAEV to ovine lentivirus (OvLV) and the interspecies transmission of these viruses necessitate comparative phylogeny of small ruminant lentiviruses [4].

Lesions suggestive of bronchointerstitial pneumonia were observed in 2 sheep (2.4%) and 3 goats (4.7%). The gross and microscopic features detected in this study were consistent with the previous reports [34]. Bronchointerstitial pneumonia happens when viral agents affect the lungs as the primary etiology of interstitial pneumonia with further invasion of the infected lungs by bacteria [34]. Bronchointerstitial pneumonia, among others, is widely accepted as the novel pathologic finding observed in natural Peste des Petits Ruminants (PPR) virus infection in sheep and goats [4, 59–61]. Several studies have reported the presence of PPR in Ethiopia particularly in pastoral areas with huge small ruminant potential [62–65].* Mycoplasma ovipneumoniae* is also incriminated as a cause of atypical or chronic progressive pneumonia in sheep with an interstitial bronchopneumonia pattern [66]. The majority of these strains were detected by dot blot test on pneumonic lung extracts and pleural exudate samples collected from respiratory cases that occurred in different regions in Ethiopia [67].

Aspiration pneumonia was detected in both sheep (4.8%) and goats (4.7 %) with equal proportions and more or less the same gross and histopathological findings. The observed lesions were similar with the reports of Caswell and Williams [4] and Antoniassi et al. [68]. The gross and microscopic lesions of aspiration pneumonia are greatly variable and usually depend on the physical and chemical nature of the aspirated material. Apart from the inherent inflammatory and necrotic nature of the aspirated fluid, most bacteria from the nasopharynx will be flushed down the respiratory tree and reach the lungs by gravitational drainage and cause lesions ranging from bronchopneumonia to granulomatous in type [69, 70]. The common conditions predisposing small ruminants to aspiration pneumonia include poor dipping techniques, faulty administration or drenching of milk or medicaments, presence of cleft palate, and some infectious and neurologic diseases causing laryngeal dysfunction [71]. Perhaps, faulty administration or drenching of medicaments took the lead because of the prevailing shortage of veterinary service in the study areas where these animals originated.

Ovine pulmonary adenocarcinoma (OPA), also known as Jaagsiekte disease, was the least form of pneumonia that was detected in only five sheep. The gross and histopathologic features observed were classic and similar to the previous reports from Ethiopia and elsewhere [36, 55, 58, 72]. Concurrent infection of OPA with suppurative bronchopneumonia and ovine progressive pneumonia was also reported earlier by Woldemeskel and Tibbo [55]. Previous reports of OPA in Ethiopia were from the central highlands of the country [55, 58]. In contrast, the current study revealed for the first time the presence of the disease in Borena (5 cases) and Arsi (1 case), which are representative of typical pastoral lowland and central plateau, respectively. The gradual spread of the disease to other parts of the country can partly be explained by the unrestricted transportation of animals from place to place and the widespread distribution of improved breeds from breeding centers located at central highlands. Owing to the long incubation period and the subclinical form of the disease, its spread to other parts of the country probably increases in multiple folds. Since the existing serological tests fail to diagnose the disease [73, 74], similar abattoir based studies are crucial to study the spatial distribution of the disease and hence take the necessary control and preventive measures.

## 5. Conclusion

In general, the current histopathological study unveiled that more than 17% of sheep and goats slaughtered at Elfora abattoir were affected with different forms of pneumonia. Bronchopneumonia and interstitial pneumonia were the major forms of pneumonia detected. As most of the pulmonary disease was diagnosed as acute while the animals were held alive in pens at the abattoir prior to slaughter, reducing this period to the minimum could significantly reduce the magnitude. Apart from its better sensitivity and specificity, the histopathologic diagnosis of lungs potentially helped to survey the various forms of pneumonia occurring in different corners of the country. Therefore, histopathology should be employed routinely as an ancillary test in the major abattoirs and regional veterinary laboratories to generate additional epidemiological data for a better disease control and prevention measures. Further studies focusing on the spatial distribution of the different pneumonic diseases and isolation of the causative agents are also required to formulate feasible and cost-effective control strategies.

## Figures and Tables

**Table 1:** Prevalence of pneumonia in the lungs of sheep and goats based on gross and histopathological examinations.

Animal	Total examined	Gross examination		Histopathological examination	
		No (%) positive	95%CI	No (%) positive	95%CI
Sheep	490	89 (18.16)	14.7-21.6	84 (17.14)	13.8-20.5
Goats	374	69 (18.45)	14.5-22.4	64 (17.11)	13.3-20.9
Total	864	158 (18.29)	15.7-20.9	148 (17.13)	14.6-19.6

**Table 2:** Types of pneumonia found in sheep and goats by histopathological diagnosis.

Types of pneumonia	Sheep (n = 84)	Goat (n = 64)	Overall (n = 148)
	No (%)	No (%)	No (%)
Interstitial pneumonia	43 (51.2)	19(29.7)	62 (41.9)
Acute suppurative bronchopneumonia	15(17.9)	23(35.9)	38 (25.7)
Acute fibrinous bronchopneumonia	16(19)	20(31.3)	36(24.3)
Chronic bronchopneumonia	7(8.3)	2(3.1)	9(6.1)
Aspiration pneumonia	4(4.8)	3(4.7)	7(4.7)
Ovine pulmonary adenomatosis	5(6)	-	5(3.4)
Broncho-interstitial pneumonia	2(2.4)	3(4.7)	5(3.4)

## Data Availability

The data used to support the findings of this study are available from the corresponding author upon request.
